# Patient Perspectives on Repeat Intubation Following Mechanical Ventilation: A Pilot Retrospective Survey Study

**DOI:** 10.7759/cureus.114649

**Published:** 2026-08-17

**Authors:** Cameron Bondy, Kendal Tapia, Abdulhaadi Khan, Janie Hu, Mufadda Hasan

**Affiliations:** 1 Internal Medicine, Arrowhead Regional Medical Center, Colton, USA; 2 Pulmonary and Critical Care Medicine, Arrowhead Regional Medical Center, Colton, USA

**Keywords:** code status, critical care, dni, do not intubate, goals of care, intensive care unit, intubation, mechanical ventilation, patient experience, patient perspective

## Abstract

Mechanical ventilation is a common life-saving intervention in critically ill patients; however, little literature exists examining how prior intubation experiences influence future patient preferences regarding repeat intubation or Do Not Intubate (DNI) status. This pilot study evaluated patient perceptions following mechanical ventilation and assessed willingness to undergo repeat intubation if medically necessary. We conducted a pilot observational survey study of adult patients previously hospitalized at a community teaching hospital who underwent mechanical ventilation identified through intubation/extubation order sets. Participants completed a structured survey evaluating awareness of intubation, understanding of the indication for intubation, recollection of the experience, perceived pain and discomfort, adequacy of sedation and pain control, and willingness to undergo repeat intubation.

Responses regarding prior consideration of DNI status and post-hospitalization code-status preferences were also collected. Twenty-seven patients completed the survey. Most participants were aware they had been intubated (81.5%), and 74.1% reported understanding why intubation was required. Intubation was described as painful by 29.6% of participants and uncomfortable by 55.6%. While 70.4% felt adequately sedated and 74.1% felt their pain was adequately controlled, recollection of the mechanical ventilation experience varied: 44.4% remembered none of their time intubated, 33.3% remembered some, 14.8% remembered most, and 7.4% remembered all of the experience. When asked whether they would agree to repeat intubation if medically necessary, 77.8% responded yes, while 11.1% responded no, 7.4% responded maybe, and 3.7% remained undecided. Prior to hospitalization, only 22.2% had previously considered or discussed DNI status. Following their hospitalization, 11.1% expressed interest in becoming Do Not Resuscitate (DNR), while 7.4% remained uncertain. Family influence on DNI-related decision-making was reported by 42.9% of respondents.

## Introduction

Mechanical ventilation is a frequently utilized life-saving intervention in critically ill patients with respiratory failure, altered mental status, shock, and other forms of acute physiologic compromise. Although endotracheal intubation is often necessary to stabilize critically ill patients, the experience of mechanical ventilation may be physically and psychologically distressing. Patients commonly report discomfort, pain, anxiety, helplessness, impaired communication, and fragmented or traumatic memories associated with intubation and intensive care unit stays [1-5]. Given these experiences, it is plausible that prior mechanical ventilation may influence patients’ future treatment preferences, including their willingness to undergo repeat intubation if medically necessary.

Despite the widespread use of mechanical ventilation, little literature exists examining how prior intubation experiences influence future code-status preferences, particularly decisions regarding Do Not Intubate (DNI) status. Prior literature has discussed the complexity of Do Not Resuscitate (DNR)/DNI decision-making and the frequent conflation of these concepts in clinical practice; however, the proportion of patients who would decline repeat intubation after experiencing mechanical ventilation remains poorly characterized [6-8]. To our knowledge, this is among the first pilot studies attempting to quantify post-intubation DNI preference following hospitalization requiring mechanical ventilation.

Understanding this potential transition toward DNI preference has important implications for goals-of-care discussions, advance care planning, patient counseling, and shared decision-making in both inpatient and outpatient settings [9-11].

The objective of this pilot retrospective survey study was to evaluate patient perceptions following mechanical ventilation and determine the proportion of previously intubated patients who would decline future intubation if medically necessary. Secondary objectives included assessing patient recollection of intubation, perceived pain and discomfort, adequacy of sedation and pain control, and prior consideration of DNI status. We hypothesized that a meaningful subset of patients would report unwillingness to undergo repeat intubation following their hospitalization experience.

## Materials and methods

We conducted a retrospective observational pilot survey study of adult patients previously hospitalized at Arrowhead Regional Medical Center, a community teaching hospital in San Bernardino, CA, USA, who underwent mechanical ventilation during their admission. Eligible participants were identified through review of electronic medical records for documented intubation and extubation order sets placed during hospitalizations occurring between 2025 and 2026. Telephone surveys were conducted following hospital discharge, with eligible patients contacted within two years of their index hospitalization.

Inclusion criteria consisted of patients aged 18 years or older with a prior hospitalization requiring endotracheal intubation and subsequent extubation. Exclusion criteria included inability to contact the patient by telephone, inability to provide verbal informed consent, incomplete survey participation, or patient death prior to follow-up interview.

Participants were identified using convenience sampling through retrospective electronic medical record review. Patients were contacted via telephone following identification through the electronic medical record. Prior to participation, verbal informed consent was obtained from all participants. All interviews and survey administration were performed exclusively by telephone.

Participants completed a structured questionnaire designed to evaluate patient perspectives regarding their prior intubation experience. The questionnaire used in this study was developed by the study authors and is included in Appendix A. Survey questions assessed awareness of intubation, understanding of the indication for intubation, recollection of the mechanical ventilation experience, perceived pain and discomfort, adequacy of sedation and pain control, and willingness to undergo repeat intubation if medically necessary in the future. Additional questions evaluated prior consideration of DNI status, potential changes in code-status preferences following hospitalization, and the influence of family members on future intubation-related decision-making. Demographic information including age and gender was also collected.

Several clinical factors, including indication for intubation, duration of mechanical ventilation, severity of illness, ICU delirium, sedation exposure, preexisting psychiatric illness, and time elapsed since hospitalization, may influence patient perceptions following mechanical ventilation. Given the pilot nature of this study and limited sample size, these variables were not collected for formal analysis and are acknowledged as limitations of the present study.

Survey responses were recorded and analyzed descriptively. Given the pilot nature of the study and limited sample size, results were summarized using frequencies, percentages, means, and ranges where appropriate. Responses indicating willingness, unwillingness, “maybe,” and “undecided” regarding future intubation were reported as separate descriptive categories. Responses of “maybe” and “undecided” were not combined with either willing or unwilling responses for analysis. No formal hypothesis testing or multivariable statistical analysis was performed. The primary outcome was patient preference regarding repeat intubation if medically necessary.

This study was approved by the Arrowhead Regional Medical Center Institutional Review Board (Protocol #26-03). The requirement for written informed consent was waived, and verbal informed consent was obtained from all participants prior to survey administration.

## Results

Twenty-seven patients completed the survey. Most participants were aware they had been intubated (22/27, 81.5%), and 74.1% (20/27) reported understanding why intubation was required. Intubation was described as painful by 29.6% (8/27) of participants and uncomfortable by 55.6% (15/27). While 70.4% (19/27) felt adequately sedated and 74.1% (20/27) felt their pain was adequately controlled, recollection of the mechanical ventilation experience varied: 44.4% (12/27) remembered none of their time intubated, 33.3% (9/27) remembered some, 14.8% (4/27) remembered most, and 7.4% (2/27) remembered all of the experience. These results are described in Table 1. 

**Table 1:** Demographic characteristics and survey responses among previously mechanically ventilated patients (n = 27). DNI: Do Not Intubate; DNR: Do Not Resuscitate

Variable	Response	n (%)
Mean age	—	51.9 years
Age range	—	21–86 years
Male sex	—	15 (55.6)
Female sex	—	12 (44.4)
Awareness of intubation	Yes	22 (81.5)
	No	5 (18.5)
Understood indication for intubation	Yes	20 (74.1)
	No	7 (25.9)
First time intubated	Yes	19 (70.4)
	No	8 (29.6)
Recollection of mechanical ventilation		
None	—	12 (44.4)
Some	—	9 (33.3)
Most	—	4 (14.8)
All	—	2 (7.4)
Patient experience during intubation		
Intubation painful	Yes	8 (29.6)
	No	19 (70.4)
Intubation uncomfortable	Yes	15 (55.6)
	No	12 (44.4)
Adequately sedated	Yes	19 (70.4)
	No	4 (14.8)
	Unsure	4 (14.8)
Adequate pain control	Yes	20 (74.1)
	No	4 (14.8)
	Unsure	3 (11.1)
Future intubation and code-status preferences		
Would undergo repeat intubation	Yes	21 (77.8)
	No	3 (11.1)
	Maybe	2 (7.4)
	Undecided	1 (3.7)
Prior consideration/discussion of DNI	Yes	6 (22.2)
	No	21 (77.8)
Interested in DNR status following hospitalization	Yes	3 (11.1)
	No	22 (81.5)
	Unsure	2 (7.4)
Family influence on intubation/DNI decisions	Yes	12 (44.4)
	No	15 (55.6)

**Figure 1:**
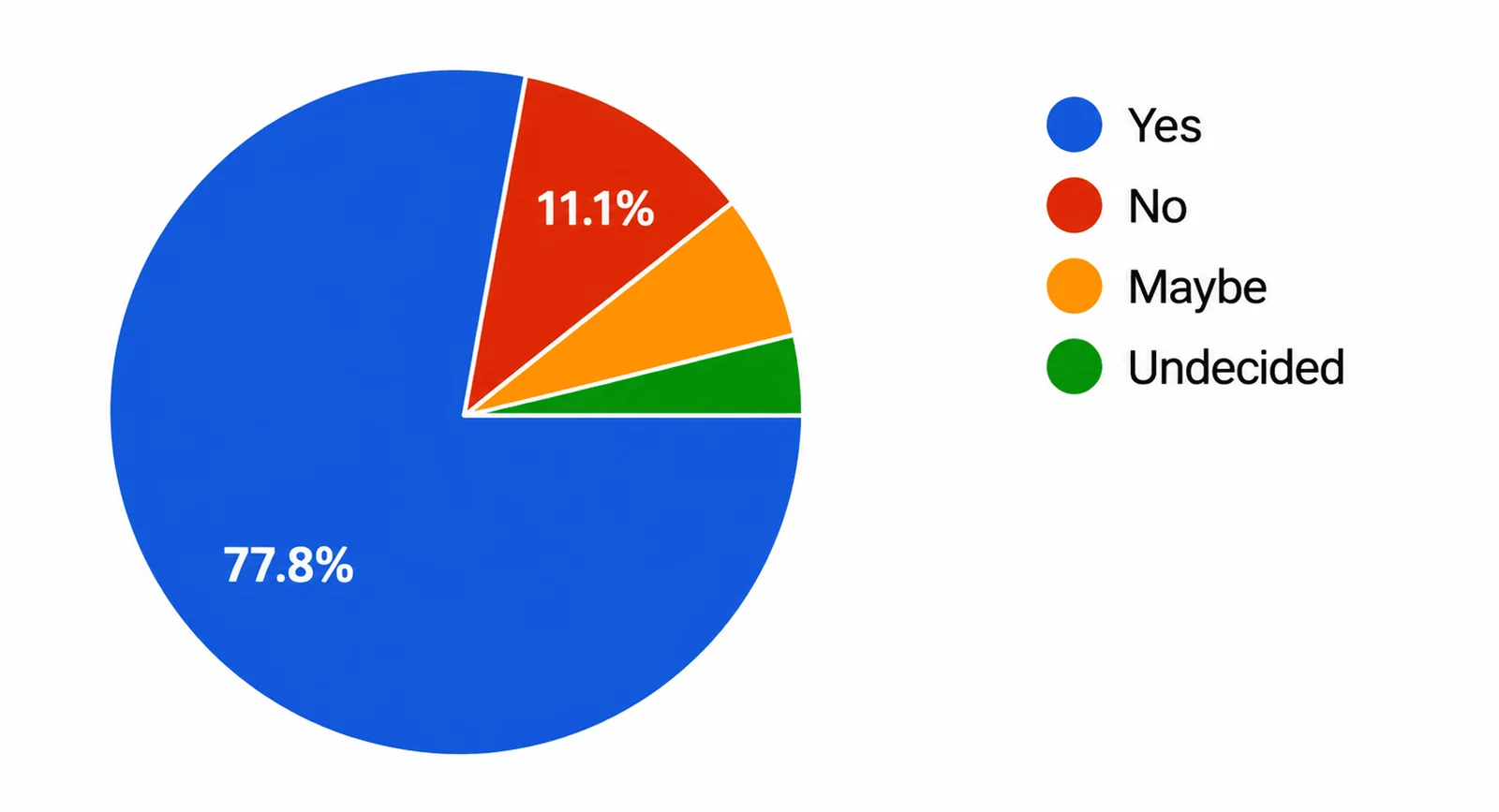
Percentage of patient's willing versus unwilling to be intubated in the future if medically necessary

The primary outcome of interest was willingness to undergo repeat intubation if medically necessary in the future. When asked whether they would agree to repeat intubation if medically necessary, 77.8% (21/27) responded yes, while 11.1% (3/27) responded no, 7.4% (2/27) responded maybe, and 3.7% (1/27) remained undecided. Prior to hospitalization, only 22.2% (6/27) had previously considered or discussed DNI status. Following hospitalization, 11.1% (3/27) expressed interest in becoming DNR, while 7.4% (2/27) remained uncertain. Family influence on DNI-related decision-making was reported by 44.4% (12/27) of respondents. These results are presented in Figure 1. 

## Discussion

This pilot study examined patient perspectives following mechanical ventilation and sought to determine the proportion of previously intubated patients who would decline future intubation. To our knowledge, there is currently limited published literature evaluating patients’ willingness to undergo repeat intubation following prior mechanical ventilation. Although decisions regarding future intubation are inherently individualized and influenced by prognosis, quality of life, and patient values, understanding patient perspectives after lived experience with mechanical ventilation may help inform these discussions. Multiple factors likely influence a patient’s consideration of DNI status, including recollection of the intubation experience, psychological sequelae following critical illness, the clinical context surrounding intubation, and family dynamics.

Interestingly, although many participants described intubation as uncomfortable or painful, most remained willing to undergo repeat intubation if medically necessary. This distinction is important because discomfort alone may not necessarily translate into refusal of future life-sustaining therapy. One possible explanation is that patients may contextualize the temporary distress of mechanical ventilation against its perceived survival benefit; however, the present study did not evaluate the reasons underlying participants’ responses, and this hypothesis warrants further investigation.

These results also suggest that patient perception of intubation may extend beyond physical discomfort alone. A psychological component may influence how patients interpret and remember their hospitalization experience. According to one study, among patients intubated for chronic obstructive pulmonary disease exacerbation, more than half recalled a feeling of suffocation while on the ventilator [12]. In another study, nearly one-third of patients mechanically ventilated for more than 48 hours described sensations of air hunger or inadequate airflow through the endotracheal tube [13]. More recent literature continues to recognize dyspnea and air hunger as important yet underrecognized symptoms among mechanically ventilated patients. These symptoms may contribute to the development of post-traumatic stress disorder following critical illness, which affects up to 20% of ICU survivors [14-15].

Similarly, patients intubated for planned surgical procedures may perceive mechanical ventilation differently than patients intubated in the setting of acute medical illness, respiratory failure, or life-threatening physiologic compromise. Surgical patients often anticipate intubation as part of a controlled perioperative process with an expected recovery, whereas medically intubated patients may experience greater fear, uncertainty, delirium, or loss of control surrounding their illness [1-5]. Further investigation comparing perceptions between surgical and medical intubation populations may help clarify these differences.

Another notable finding was that relatively few participants had considered or discussed DNI status prior to their hospitalization. This highlights the potential role of severe illness and ICU admission as catalysts for future goals-of-care discussions and advance care planning [9-11]. Even among patients who remained willing to undergo repeat intubation, the experience itself may prompt greater reflection regarding advance care planning and future medical decision-making.

Importantly, survivorship and selection bias may have influenced the findings of this study. All surveyed participants survived hospitalization and were well enough to participate in telephone interviews following discharge. Additionally, patients who could not be reached by telephone or declined participation may have differed systematically from those who completed the survey. Patients who experienced the most severe physical or psychological distress related to intubation may have been underrepresented because they did not survive to discharge, were unable to participate, or declined follow-up interviews. Consequently, the true proportion of patients who would decline future intubation following mechanical ventilation may be higher than observed in this cohort.

This study has several important limitations. The sample size was relatively small and derived from a single-center retrospective cohort, which may limit generalizability. Additionally, the retrospective survey design introduces potential recall bias, and patient experiences with mechanical ventilation are influenced by numerous clinical factors, including the indication for intubation, duration of mechanical ventilation, sedation practices, illness severity, delirium, neurological recovery, and overall hospitalization experience, many of which were not captured in this pilot analysis. Furthermore, nearly one-third of participants had undergone prior intubation before the index hospitalization. Consequently, their responses may have reflected cumulative experiences with mechanical ventilation rather than the index hospitalization alone. Larger prospective studies incorporating these variables and adequately powered to evaluate associations between patient experiences and future treatment preferences are needed to better characterize patient perceptions of mechanical ventilation and identify factors associated with future code status preferences.

## Conclusions

In this pilot retrospective survey study, most previously intubated patients reported willingness to undergo repeat mechanical ventilation if medically necessary, despite many describing the experience as uncomfortable or painful. Contrary to our initial expectation, relatively few participants expressed unwillingness to undergo repeat intubation following their hospitalization. These findings suggest that prior experiences with mechanical ventilation do not necessarily translate into unwillingness to undergo future life-sustaining therapy and underscore the importance of individualized goals-of-care discussions that consider each patient’s values, prior experiences, and clinical circumstances. Larger prospective studies are needed to further characterize the factors influencing future treatment preferences following mechanical ventilation.

## Figures and Tables

**Figure 2:**
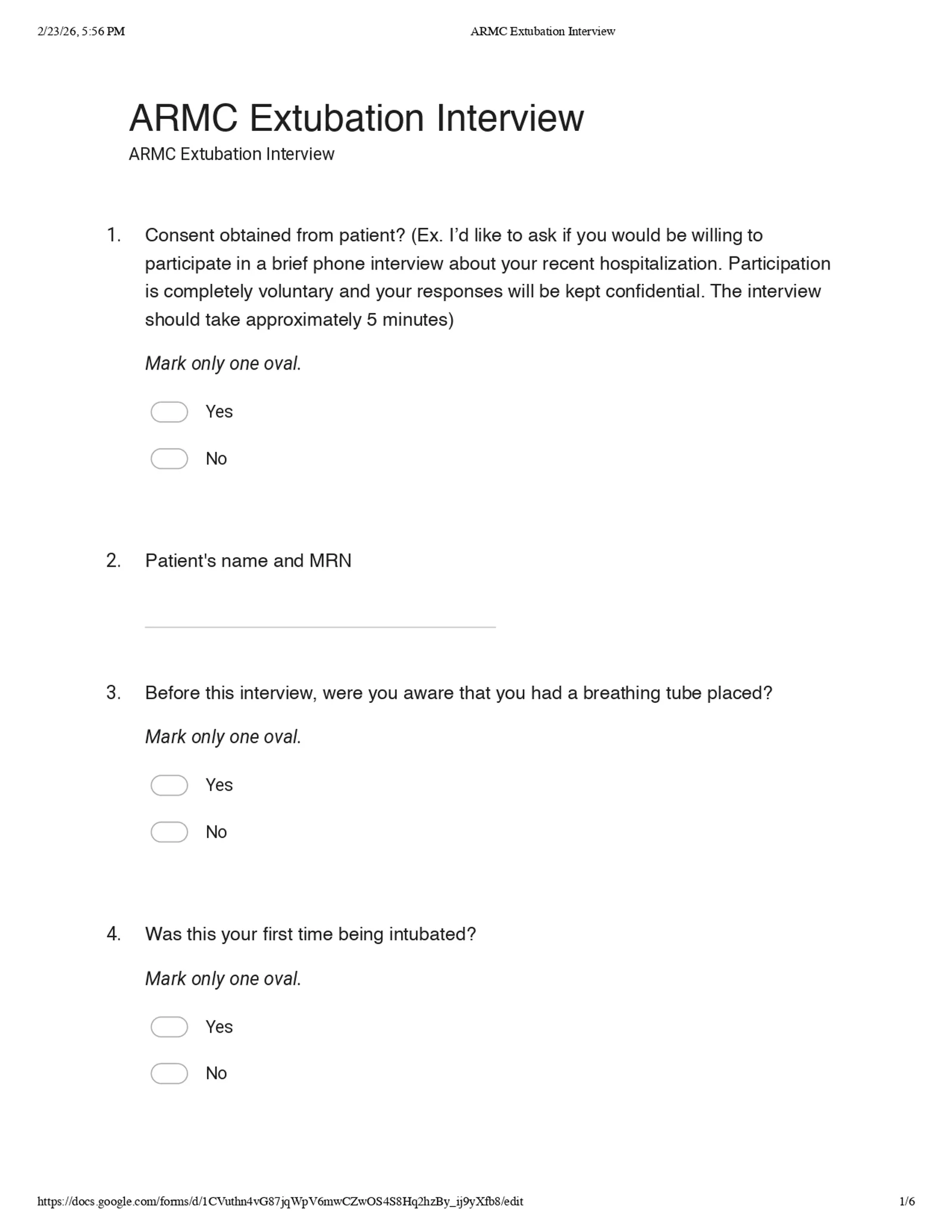
Questionnaire: part 1

**Figure 3:**
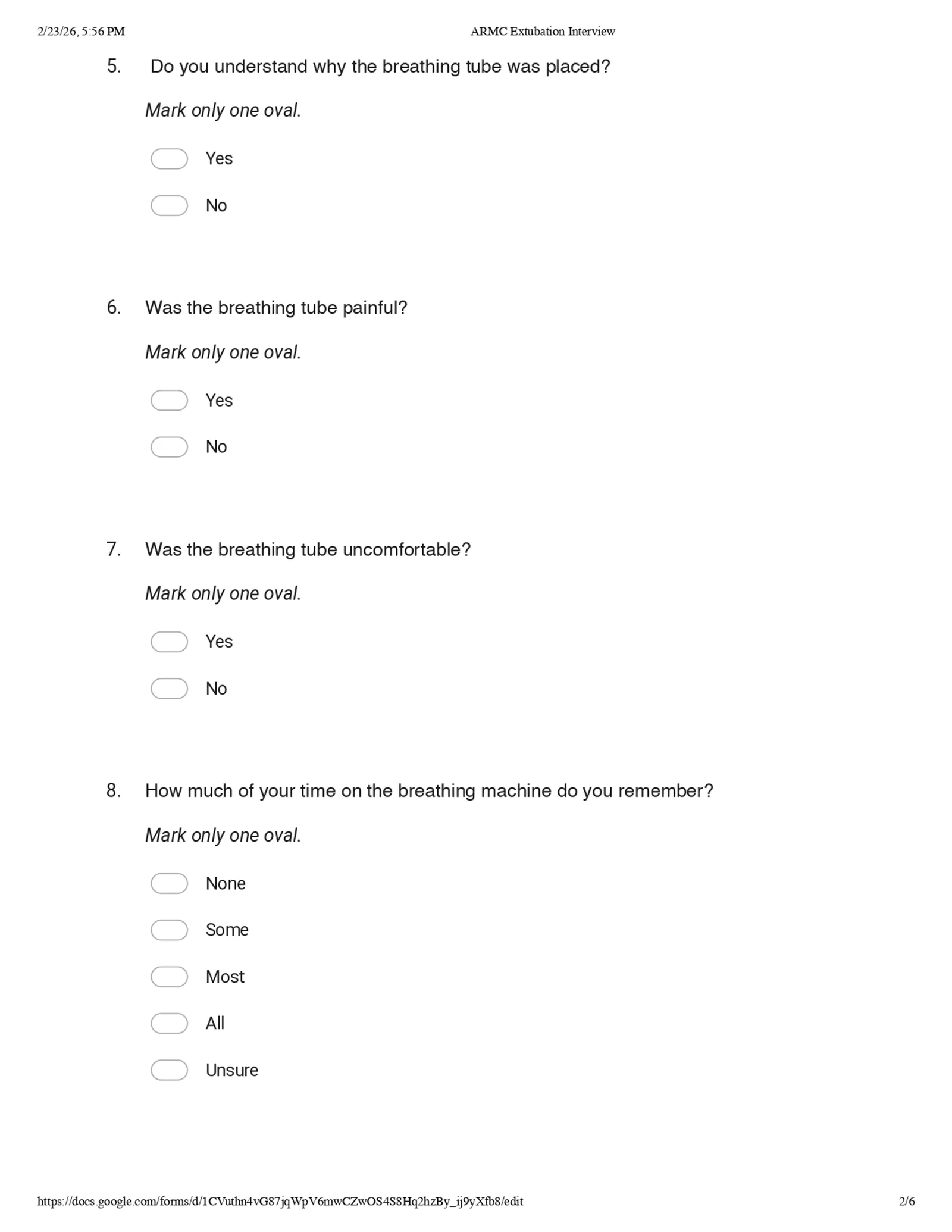
Questionnaire: part 2

**Figure 4:**
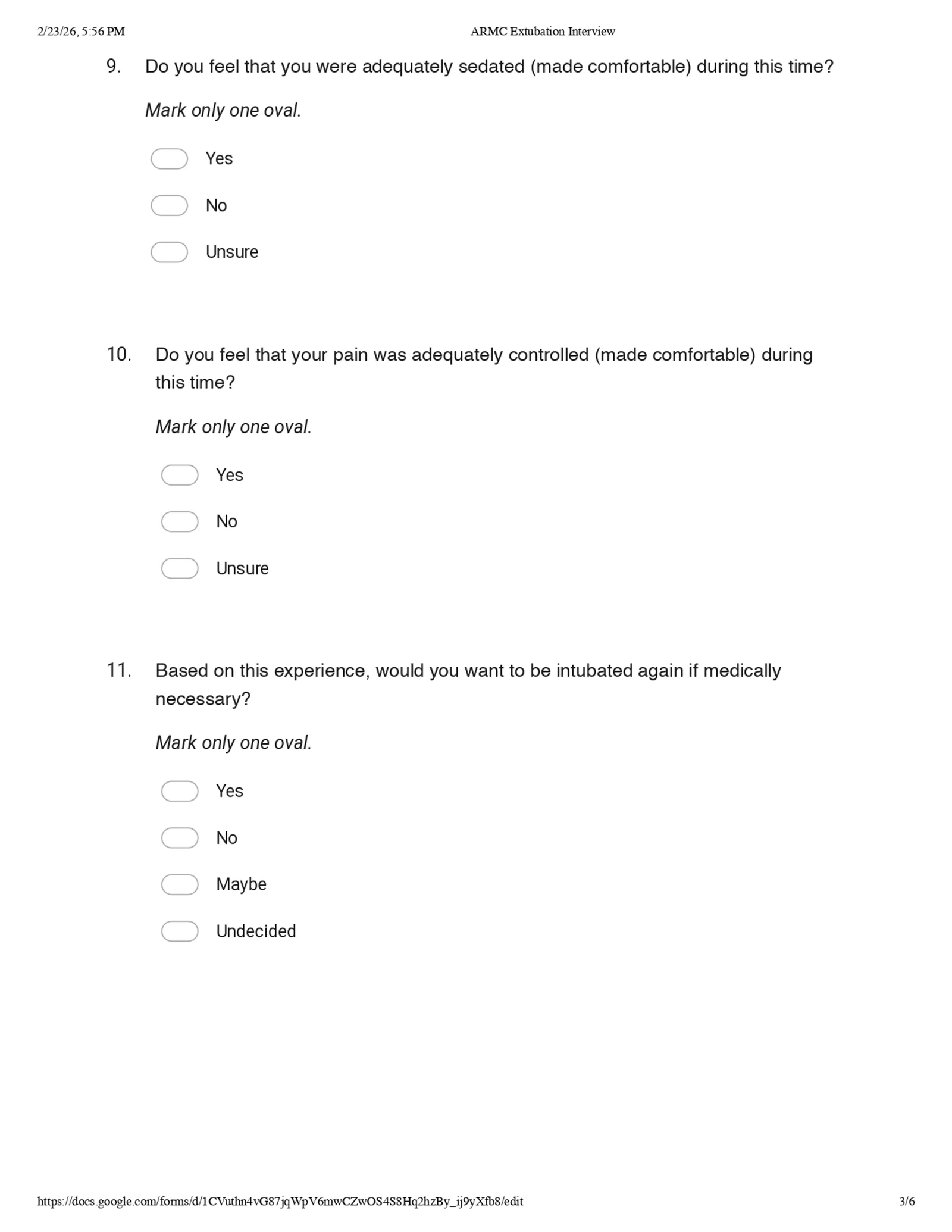
Questionnaire: part 3

**Figure 5:**
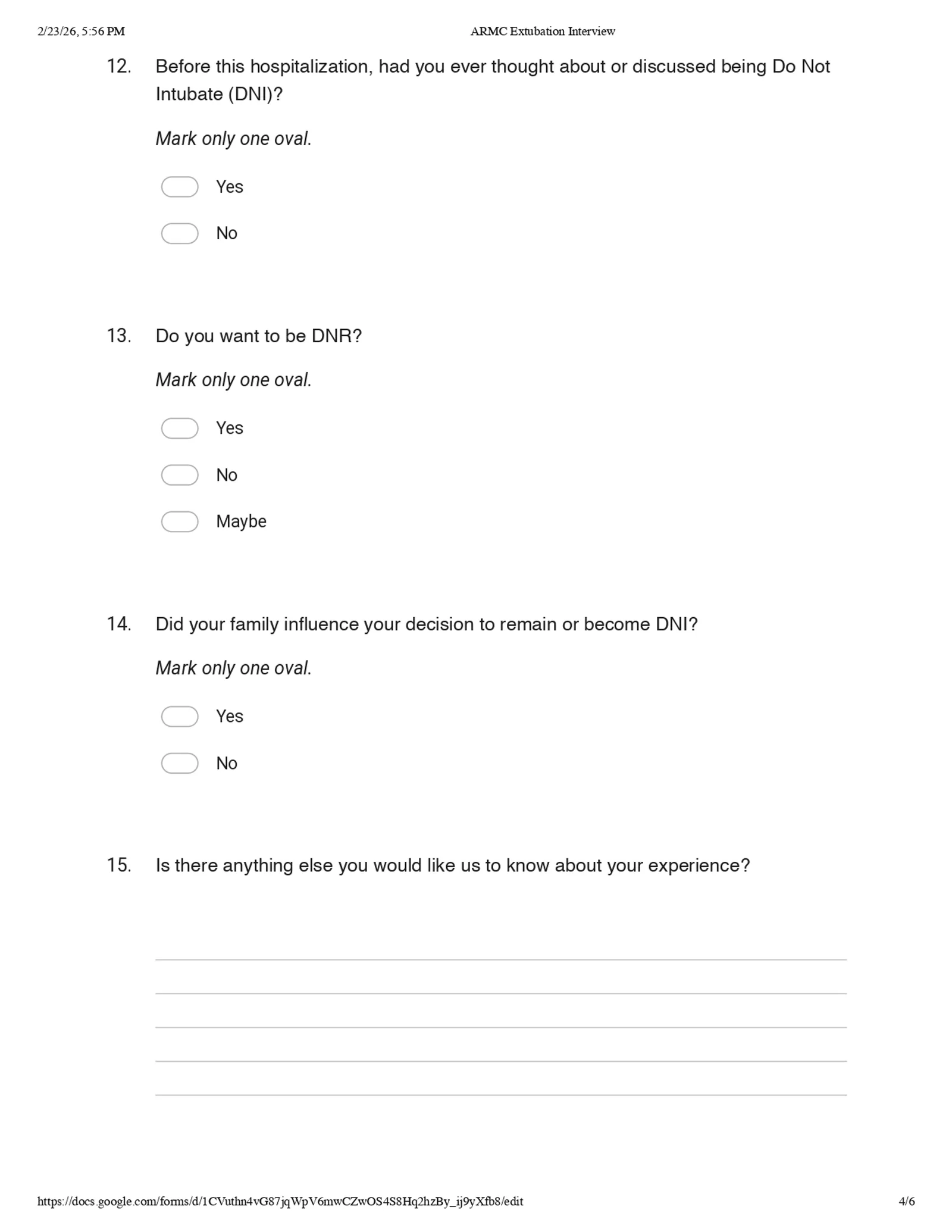
Questionnaire: part 4

## Appendices

Appendix A

Study Questionnaire
